# Editorial: Typical and Atypical Processing of Gaze

**DOI:** 10.3389/fpsyg.2019.02576

**Published:** 2019-11-19

**Authors:** Chris Ashwin, Paola Ricciardelli

**Affiliations:** ^1^Centre for Applied Autism Research, Department of Psychology, University of Bath, Bath, United Kingdom; ^2^Department of Psychology, University of Milano-Bicocca, Milan, Italy

**Keywords:** eyes, gaze processing, gaze direction, mental states, attention, autism spectrum disorder

Eye gaze represents a central source of socially meaningful cues which helps convey key information about the focus of others attention. Gaze direction is used for non-verbal communication, which includes making inferences about the mental states, intentions, beliefs, and desires of other people. The direction of gaze is such an automatic and powerful cue that it triggers the observers' own attention in the same direction, even when that information is uninformative, and allows individuals to build up shared representation of the inner and outside world.

Despite the importance of gaze processing in the social world we still know surprisingly little about how we process this information, including the cognitive and neural mechanisms and the factors affecting gaze processing. This includes about how gaze processing is different in various disorders with difficulties in social cognition, such as autism, which is reported to involve reduced expertise for perceiving gaze direction (Ashwin et al., 2009). The contributions of this Research Topic have utilized various research methodologies and techniques testing the effects of different factors on gaze processing toward a better understanding about the underlying mechanisms across different types of samples e.g., typical controls, brain-damaged patients or those with various disorders. The studies in this eBook also test how we integrate the different types of information conveyed by gaze with the context and our environment. The studies and reviews are briefly summarized along with some remarks about their contributions.

## Factors Affecting Gaze Processing in Typical Samples

A number of factors related to gaze processing were investigated by the studies reported, with the first section including studies involving typical control participants. The role of situational factors was examined using an innovative experimental design in the study by Balsdon et al., where participants were asked either to make a left/right gaze judgement (single task—directional) or direct/indirect gaze judgement (two-interval task—non-directional). Results showed that observers integrated the same sensory information from head orientation and gaze direction in a flexible manner, giving them a different weight depending on the kind of judgment made in-line with previous studies (e.g., Ricciardelli and Driver, 2008). The effects of eccentricity of gaze processing was tested in a study by Yokoyama and Takeda, where they investigated whether the gaze cuing effect requires foveal vision. Results showed that this effect occurs even when the gaze stimuli appear in peripheral vision up to 5° of eccentricity from the attentional focus. The study by Awad et al. tested the importance of the context and the emotional expressions of the face being judged on gaze processing, reporting that the perception of gaze direction depended on both the face eccentricity and its emotion. Together, these studies demonstrate that you do not need to be looking directly at someone for their gaze to affect your attention, which may be how gaze perception is often done in real-life social situations.

Tipples et al. investigated whether a gaze-liking effect, which involves an increase in the likeability of objects which have been repeatedly gazed at (e.g., Bayliss et al., 2006), can also occur for verbal descriptions of looking behavior without the need of attentional shifts. The authors compared the effect of pointing gestures and arrow cues and the results provide partial support for the gaze specificity hypothesis. However, the liking effect was enhanced for gaze cues compared to arrows indicating that eye gaze is a highly effective cue. The effect of eye contact in memory recognition has been shown in the study by Lanthier et al., who manipulated several contextual factors (i.e., live eye contact, the temporal dynamics and the communicative meaning of the gaze, and gender) during a word memory recognition task and reported that eye contact improves recognition, but only in females. A study by Capellini et al. showed that experiencing social exclusion alters joint attention behavior. They used the *Cyberball* manipulation to induce feelings of ostracism. The socially excluded participants had reduced attentional shift toward averted gaze than those who were included, although pointing arrows elicited an attentional orienting response in both groups. This may be because the ostracized participants perceive averted gaze as a further sign of exclusion. These last three studies showed that gaze processing can modify other cognitive processes such as word processing and memory and even judgments about the self.

The effects of task demands were investigated by McCrackin and Itier who measured EEG during tasks of emotion recognition, direction of attention, and gender discrimination involving images of individuals with direct or averted gaze. They report differences in accuracy and also neural processing as measured by ERP's, based on whether the gaze in the individuals being perceived was direct or averted across the tasks. The article by Jarick and Bencic measured physiological arousal during a live task with dyads of strangers and found greater arousal when both participants were making eye contact with each other (i.e., both sending and receiving gaze information), compared to other conditions where only one person was sending/receiving gaze information or no gaze information was communicated between them. Both the studies described in this paragraph helped reveal about the underlying neural and physiological effects of gaze direction and eye contact.

## Typical and Atypical Gaze in Disorders and Patients

Lopis and Conty investigated whether there might be enhanced memory for face-name associations in people with Alzheimer's disease when images of other individuals contain direct gaze than averted gaze, based on previous reports of direct gaze improving memory for subsequent faces and verbal information. They did not find enhanced memory for face-name associations in Alzheimer patients, but across all participants, they found that direct gaze context produced greater memory for faces and for names presented independently. Therefore, some gaze effects are preserved in both normal aging and Alzheimer's disease. A study by Tsuji and Shimada used psychophysics measures to investigate the impact of socially anxious tendencies on gaze perception. They tested the presence of a negative bias when judging the valence (positive or negative) of gaze in emotionally ambiguous faces using only the eye region of either disgusted, happy or neutral faces. They found that lower negative intensity ratings of gaze were associated with higher social anxiety scores, suggesting that those with higher phobia tendencies experience more anxiety toward the gaze of others, likely because they perceive it as threatening.

The importance of eye gaze in driving attention was examined in a sample of patients with unilateral neglect in the study by Rato et al. The authors used a modified version of a cancellation test, where the targets to be canceled were direct gaze or averted gaze stimuli appearing among the distracters with closed eyes. Their findings speak in favor of the effectiveness of direct gaze to capture attention and improve neglect, which is characterized by an attentional orienting deficit. The article by Del Bianco et al. included an eye-tracking paradigm used to investigate attention to faces in adults with and without autism spectrum disorder (ASD), and reports that people with ASD showed initial attention to faces comparable to controls, and they had even greater fixation times on bodies and faces than controls, which is contrary to what is typically reported and theorized in ASD but may reflect greater processing demands of social information which require greater attention. Interestingly, those with ASD were more influenced than controls by the different task instructions by showing variable responses across conditions than controls. These studies on pathological and atypical individuals showed the importance of gaze perception in humans because many of the samples showed intact processing of the gaze of others and typical effects of gaze on other cognitive processes.

## Reviews About Gaze Processing

Three of the contributions to the Research Topic were literature reviews about gaze processing. Clifford and Palmer provide a review about the phenomenon of adaptation to the gaze direction of others, which is a perceptual aftereffect that occurs after viewing someone gazing in a specific direction for a long period of time (i.e., after viewing a specific gaze direction subsequent gaze directions are perceived differently). They report about studies investigating whether the effect occurs at higher or lower levels of processing and proposed potential mechanisms of adaptation and gaze coding, and how gaze adaptation is reported to be different in autism. Hietanen reviews the evidence from studies reporting about the effects of eye contact on affective reactions, and proposes that the physiological and cognitive reactions of emotional arousal are clearer for studies involving the implicit processing of eye contact compared to those involving explicit processing and offers some interesting ideas about the potential underlying mechanisms. A review by Cañigueral and Hamilton highlights the dual functioning of eye gaze for having roles in both perceiving social information and signaling social information to others, and with the roles being more evident during face-to-face interactions over typical lab experiments using only one mode of communication (e.g., a participant viewing a computer screen with image of someone gazing). They propose the Interpersonal Gaze Processing model and discuss differences in interactive gaze functioning in autism. Together, these reviews give a new and updated view of the literature about the neural, physiological and cognitive mechanisms of gaze processing and provide some directions for future research.

## Author Contributions

All authors listed have made a substantial, direct and intellectual contribution to the work, and approved it for publication.

### Conflict of Interest

The authors declare that the research was conducted in the absence of any commercial or financial relationships that could be construed as a potential conflict of interest.
